# Combined PET/MRI: *Global Warming—*Summary Report of the 6th International Workshop on PET/MRI, March 27–29, 2017, Tübingen, Germany

**DOI:** 10.1007/s11307-017-1123-5

**Published:** 2017-10-02

**Authors:** D. L. Bailey, B. J. Pichler, B. Gückel, G. Antoch, H. Barthel, Z. M. Bhujwalla, S. Biskup, S. Biswal, M. Bitzer, R. Boellaard, R. F. Braren, C. Brendle, K. Brindle, A. Chiti, C. la Fougère, R. Gillies, V. Goh, M. Goyen, M. Hacker, L. Heukamp, G. M. Knudsen, A. M. Krackhardt, I. Law, J. C. Morris, K. Nikolaou, J. Nuyts, A. A. Ordonez, K. Pantel, H. H. Quick, K. Riklund, O. Sabri, B. Sattler, E. G. C. Troost, M. Zaiss, L. Zender, Thomas Beyer

**Affiliations:** 1 Department of Nuclear Medicine, Royal North Shore Hospital, and Faculty of Health Sciences, University of Sydney, Sydney, Australia; 2 Werner Siemens Imaging Center, Department of Preclinical Imaging and Radiopharmacy, Eberhard-Karls-Universität, Tübingen, Germany; 3 Department of Diagnostic and Interventional Radiology, University of Tübingen, Tübingen, Germany; 4 Department of Diagnostic and Interventional Radiology, Medical Faculty, University Dusseldorf, 40225 Dusseldorf, Germany; 5 Department of Nuclear Medicine, University Hospital Leipzig, Leipzig, Germany; 6 Division of Cancer Imaging Research, Department of Radiology, The Johns Hopkins University School of Medicine, Baltimore, MD 21205 USA; 7 Praxis für Humangenetik Tübingen, Paul-Ehrlich-Str. 23, 72076 Tübingen, Germany; 8 Molecular Imaging Program at Stanford (MIPS) and Bio-X, Department of Radiology, Stanford University School of Medicine, Stanford, CA USA; 9 Department of Internal Medicine I, Eberhard-Karls University, Tübingen, Germany; 10 Department of Nuclear Medicine and Molecular Imaging, University of Groningen, University Medical Center Groningen, Groningen, The Netherlands; 11 Institute of Diagnostic and Interventional Radiology, Klinikum rechts der Isar, Technische Universität München, Munich, Germany; 12 Diagnostic and Interventional Neuroradiology, Department of Radiology, Eberhard Karls University, Hoppe-Seyler-Straße 3, 72076 Tübingen, Germany; 13 Cancer Research UK Cambridge Institute, Li Ka Shing Centre, Robinson Way, Cambridge, CB2 0RE UK; 14 Department of Biochemistry, University of Cambridge, Tennis Court Road, Cambridge, CB2 1GA UK; 15 Department of Biomedical Sciences, Humanitas University, Milan, Italy; 16 Department of Nuclear Medicine, Humanitas Research Hospital, Milan, Italy; 17 Department of Radiology, Nuclear Medicine and Clinical Molecular Imaging, Eberhard-Karls-Universität, Tübingen, Germany; 18 Department of Cancer Imaging and Metabolism, H. Lee Moffitt Cancer Center and Research Institute, Tampa, FL 33621 USA; 19 Cancer Imaging, School of Biomedical Engineering & Imaging Sciences, King’s College London, London, UK; 20 Department of Radiology, Guy’s & St Thomas’ Hospitals London, London, UK; 21 GE Healthcare GmbH, Beethovenstrasse 239, Solingen, Germany; 22 Division of Nuclear Medicine, Department of Biomedical Imaging and Image-Guided Therapy, Medical University of Vienna, Vienna, Austria; 23 New Oncology GmbH, Köln, Germany; 24 Neurobiology Research Unit, Rigshospitalet and Faculty of Health and Medical Sciences, University of Copenhagen, Copenhagen, Denmark; 25 III. Medical Department, Klinikum rechts der Isar, Technische Universität München, Munich, Germany; 26 Department of Clinical Physiology, Nuclear Medicine and PET, Rigshospitalet, University of Copenhagen, Copenhagen, Denmark; 27 Knight Alzheimer Disease Research Center, Washington University School of Medicine, St Louis, MO USA; 28 Nuclear Medicine & Molecular Imaging, KU Leuven, Leuven, Belgium; 29 Department of Pediatrics, Center for Infection and Inflammation Imaging Research, Johns Hopkins University School of Medicine, Baltimore, MD USA; 30 Institute of Tumor Biology, University Medical Center Hamburg-Eppendorf, Hamburg, Germany; 31 High Field and Hybrid MR Imaging, University Hospital Essen, Essen, Germany; 32 Erwin L. Hahn Institute for MR Imaging, University of Duisburg-Essen, Essen, Germany; 33 Department of Radiation Sciences, Umea University, Umea, Sweden; 34 OncoRay—National Center for Radiation Research in Oncology, Dresden, Germany; 35 Institute of Radiooncology—OncoRay, Helmholtz-Zentrum Dresden-Rossendorf, Dresden, Germany; 36 Department of Radiotherapy, University Hospital Carl Gustav Carus and Medical Faculty of Technische Universität Dresden, Dresden, Germany; 37 German Cancer Consortium (DKTK), Partner Site Dresden, Dresden, Germany; 38 High Field Magnetic Resonance, Max Planck Institute for Biological Cybernetics, Tübingen, Germany; 39 Department of Internal Medicine VIII, University Hospital Tübingen, Tübingen, Germany; 40 QIMP Group, Center for Medical Physics and Biomedical Engineering General Hospital Vienna, Medical University Vienna, 4L, Waehringer Guertel 18-20, 1090 Vienna, Austria

**Keywords:** PET/MRI, MR-PET, Hybrid imaging, Molecular imaging, PET/CT, PET, MRI, Quantification, Infection, Inflammation, Oncology, Neurology, Multi-parametric imaging

## Abstract

The 6th annual meeting to address key issues in positron emission tomography (PET)/magnetic resonance imaging (MRI) was held again in Tübingen, Germany, from March 27 to 29, 2017. Over three days of invited plenary lectures, round table discussions and dialogue board deliberations, participants critically assessed the current state of PET/MRI, both clinically and as a research tool, and attempted to chart future directions. The meeting addressed the use of PET/MRI and workflows in oncology, neurosciences, infection, inflammation and chronic pain syndromes, as well as deeper discussions about how best to characterise the tumour microenvironment, optimise the complementary information available from PET and MRI, and how advanced data mining and bioinformatics, as well as information from liquid biomarkers (circulating tumour cells and nucleic acids) and pathology, can be integrated to give a more complete characterisation of disease phenotype. Some issues that have dominated previous meetings, such as the accuracy of MR-based attenuation correction (AC) of the PET scan, were finally put to rest as having been adequately addressed for the majority of clinical situations. Likewise, the ability to standardise PET systems for use in multicentre trials was confirmed, thus removing a perceived barrier to larger clinical imaging trials. The meeting openly questioned whether PET/MRI should, in all cases, be used as a whole-body imaging modality or whether in many circumstances it would best be employed to give an in-depth study of previously identified disease in a single organ or region. The meeting concluded that there is still much work to be done in the integration of data from different fields and in developing a common language for all stakeholders involved. In addition, the participants advocated joint training and education for individuals who engage in routine PET/MRI. It was agreed that PET/MRI can enhance our understanding of normal and disrupted biology, and we are in a position to describe the *in vivo* nature of disease processes, metabolism, evolution of cancer and the monitoring of response to pharmacological interventions and therapies. As such, PET/MRI is a key to advancing medicine and patient care.

## Introduction

The 6th annual positron emission tomography (PET)/magnetic resonance imaging (MRI) workshop in the university town of Tübingen, Germany, was held over March 27–29, 2017. The initial Tübingen workshop in 2012 was the first of its kind to be specifically devoted to addressing methodological, clinical and research aspects of hybrid imaging using PET/MRI [1]. Attendees at the current workshop came from virtually all continents with the majority originating from Europe and only one in five having attended past workshops.

In the time since the previous workshop of 2016, the use of PET/MRI has continued to expand at a similar rate as it has done since its introduction [1–5]. To say this in a different way would be that the installation rate of new PET/MRI systems has not been as remarkable as that previously witnessed with the initial introduction of PET/X-ray computed tomography (CT). Instead, barriers to installing PET/MRI remain significant: the capital cost of the equipment, high recurrent operating costs, lack of appropriately trained staff to operate and interpret the PET/MRI studies and the lack of an evidence base demonstrating a proven role for this form of hybrid imaging in clinical use. The past 12 months have, however, seen accelerating expansion in Asia in particular, and hence, the use of the term *global warming* for this year’s summary descriptor to capture the mood of the workshop as PET/MRI is now truly a global tool with increasing use, especially clinically, thus reflective of a general “warming” of the imaging community to the value of the technology. Another factor aiding the continuing increase in acceptance of PET/MRI is that the design of the systems, which originally came in a variety of configurations, appears now to have settled on one in which the two modalities are fully integrated in a single gantry thus permitting simultaneous acquisitions with both modalities.

At times, debate at previous Tübingen workshops was dominated by issues that were considered to be technically deficient or compromised in the early systems, such as how to use MRI-based image sequences to produce appropriate correction maps to compensate for photon attenuation in the PET images [2, 3]. As long as issues such as these remained unanswered, it was difficult to focus on developing future concepts for maximally exploiting PET/MRI. It was agreed at this workshop that this particular issue was finally solved to a level of accuracy sufficient for the majority of clinical applications and/or to a level comparable to that seen in PET/CT, *i.e.* overall uncertainties seen with PET/MRI are no worse than those seen in PET/CT and were considered to be clinically acceptable. One participant during the Physics and Instrumentation dialogue board even called the topic of MRI-based attenuation correction a “case closed”, if only for applications of PET/MRI of the adult brain in the intact skull. Yet, it needs to be acknowledged that as with PET/CT, a careful inspection of PET/MRI image quality and quantification remains warranted. Hence, it was noticeable at this year’s meeting that more discussion was devoted to future applications of PET/MRI than previously, with a desire to begin “going deeper” into the interrogation of the information contained in the images.

As in the previous meetings’ reports, we will attempt to provide succinct summaries of highlight lectures, dialogue boards and major outcomes of the discussion boards. Likewise, we will highlight progress achieved, or lack thereof, in specific areas. Finally, we will adhere again to the general conventions of previous reports to indicate progress (↑), steady state (↔) and regression (↓) in key aspects of PET/MRI. The key to the summary tables of changes in PET/MRI with respect to the status of the previous year is shown in Table 1.

**Table 1:** Key to current status of PET/MRI

↑	Documented evidence of improvement in science and methodology
↗	Suggestion of improvement in methodology, but requires further investigation
↔	No change, but satisfactory status since previous workshop
↘	Little advancement in science and methodology despite previous recognition of need for improvement
↓	Less clear evidence than previously suggested

Finally, as the style of the lectures and discussions was slightly changed for this year’s workshop in an attempt to venture into detailed discussions of selected applications of PET/MRI, we will not follow the previous convention of detailing new evidence, which has emerged and future challenges in each area, but rather we will attempt to capture the recurring themes that emerged during the discussions.

## Highlight Lectures

### Highlight Lecture 1: PET/MRI Workflow

The 2017 meeting started with an invited presentation on the considerations and challenges when developing optimised workflows for PET/MRI. There are a number of key considerations for operating a PET/MRI system clinically. First, the economics/logistics are much more challenging than for PET/CT. Second, PET/MRI scans are far more demanding on readers. Finally, the scans are more expensive than PET/CT. The lecture suggested some niche clinical roles for PET/MRI and compared it to stand-alone imaging with the other modalities (Table 2).

**Table 2:** Suggested current clinical roles for stand-alone CT, PET and MRI (courtesy of A Beer, Würzburg)

CT	Robust, rapid whole-body assessment
PET	“Problem solving” whole-body tool
MRI	“Problem solving” specific regional imaging tool

It was suggested that the workflows that have been developed for PET/MRI in neurology and cardiology are straightforward [6]. A simple neurological PET/MRI examination with 2-deoxy-2-[^18^F]fluoro-D-glucose ([^18^F]FDG) can be completed in 20 min, whilst a comprehensive multi-parametric protocol using a F-18-labelled amyloid ligand for the assessment of dementia might require at least 45 min; however, all imaging is captured in a single session. Similarly, in cardiology, a complete examination can be achieved in around 45 min, as the imaging of a single organ is ideally suited to PET/MRI. An example of the complementary nature of PET and MRI in cardiology would be to use [^18^F]FDG to assess myocardial viability whilst using MRI to assess perfusion and other parameters (wall motion, *etc*) [3], or to use absolute PET perfusion measurements in conjunction with scar tissue evaluation from late enhancement MRI.

The workflows in oncology, however, are rather challenging and it may be that for adult patients PET/MRI imaging could be restricted to studying primary (target) lesions, a single body region (*e.g.,* thorax, abdomen, pelvis) or organ (*e.g.*, brain, heart, liver, pancreas) using “PET/CT guidance”. As a consequence of lengthy MR protocols resulting in longer available time to acquire the PET scan, the amount of radioactivity administered could be reduced (and hence decrease radiation exposure to the subject), or the adoption of continuous list-mode dynamic acquisitions and subsequent modelling and parametric PET image generation. As one of the main roles for PET/CT in oncology today is in staging the extent of disease for metastasis (M) and nodal spread (N)—and less so for primary tumour (T) staging—it may be that the role which emerges for PET/MRI reverts more to characterising the primary tumour, perhaps using non-FDG radiopharmaceuticals. For example, one could conceive of a protocol where a dynamic PET/MRI scan is acquired from injection of the radiopharmaceutical up to 30–60 min over the region containing the primary tumour, to capture all of the MRI sequences of interest and the PET kinetics of uptake in the primary lesion, and then the patient is taken for a conventional whole-body staging PET/CT scan commencing 60–90 min after injection to complete the metastasis and nodal evaluation. This concept has been proposed as early as 2009 by Hicks and Lau [7]. Reiterating discussions from previous Tübingen workshops, the use of DWI-MRI in patients with [^18^F]FDG avid primaries and lymph nodes was considered obsolete [4]; however, MRI, including DWI, expresses a high sensitivity and specificity for small liver lesions even though the [^18^F]FDG-PET may be negative.

### Highlight Lecture 2: Image-Guided Radiotherapy

The second highlight lecture was dedicated to the value of image-guided radiation therapy. Over the past years, wide-bore PET/MR systems that can serve the demands of the dedicated radiotherapy equipment have been designed and are being used. In this lecture, the value of [^18^F]FDG-PET for individualised target volume delineation, *e.g.* in lung cancer patients, was briefly reviewed. Moreover, the use of non-[^18^F]FDG radiotracers in radiation treatment planning (RTP) and monitoring of response was emphasised. Clinically, the ability to monitor hypoxia and re-oxygenation during combined radio-chemotherapy offers the radiation oncologist greater insight into potential response during treatment, which should ultimately lead to better outcomes.

Prospective clinical studies personalising treatment on the basis of hypoxia-PET readout are being designed or have started patient accrual. 3′-Deoxy-3′-[^18^F]fluorothymidine ([^18^F]FLT) depicting another relevant tumour characteristic in radiotherapy, *i.e.* tumour cell proliferation, was shown to be of predictive value in head and neck squamous cell carcinoma patients; however, the limited availability of the tracer has hindered its wider adoption in larger clinical trials and ultimately clinical routine. In theory, [^18^F]FDG-PET, the hypoxia PET-tracers [^18^F]fluoromisonidazole ([^18^F]FMISO) or 3-[^18^F]fluoro-2-(4-(2-nitro-1H-imidazol-1-yl)methyl) ([^18^F]HX4), and [^18^F]FLT-PET may be of additive value in defining the biological target volume. Furthermore, the international phase II clinical “PET BOOST” study in advanced stage non-small cell lung cancer (ClinicalTrials.gov identifier: NCT01024829) was mentioned, in which an enhanced “boost dose” is delivered to either the [^18^F]FDG-PET avid tumour sub-volumes or to the entire gross tumour volume whilst maintaining the dose to the surrounding organs at risk (NCT01024829). Moreover, PET/MR imaging is increasingly being utilised as an objective measure for normal tissue toxicity, *e.g.* radiation-pneumonitis in lung cancer patients [8] or neurocognitive decline in primary brain tumour patients, or as an indirect indicator of primary tumour response, *e.g.* in HNSCC [9]. A final word of caution addressed the necessity of geometrically accurate MR images in the era of image-guided high-precision radiotherapy.

**Fig. 1.**
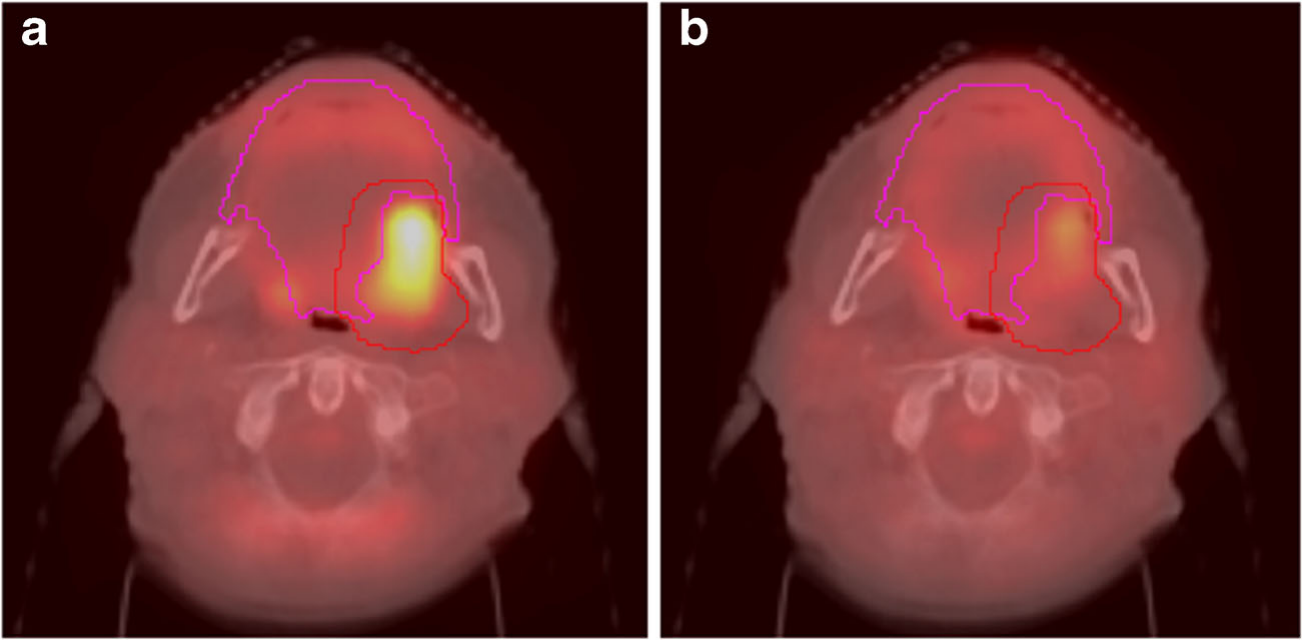
Patient with oropharyngeal carcinoma pre- and post-RT. **a** [^18^F]FDG-PET/CT prior to RT. Oral mucosa delineated in pink, clinical target volume (CTV) in red. **b** [^18^F]FDG-PET/CT during week 4 of RT. For the analysis, the CTV was subtracted from mucosa. ( Courtesy of S. Zschaeck, MD, Charité Berlin)

In conclusion, PET/MRI appears to have a number of potential uses in radiotherapy, including the adoption of integrated functional imaging to monitor response and adapt during treatments when significant changes are observed (Fig. 1). However, cross-specialty studies are needed that specifically employ radiotracers other than standard [^18^F]FDG for both the primary tumour as well as normal tissue.

### Highlight Lecture 3: Imaging the Tissue Microenvironment

The final highlight lecture presented a *tour de force* review of fundamental discoveries related to cancer at the cellular level which has been suggested as a future topic of interest for advanced PET/MRI: the assessment of tumour microenvironment. It was proposed that PET/MRI might be able to examine similar features seen in the tumour microenvironment scaled up to the whole organ or organism level. Some of the parameters discussed could be implemented already today, since they are based on existing imaging biomarkers, such as hypoxia PET agents (*e.g.* [^18^F]FMISO) or the imaging of collagen fibres, that can act as a “highway” along which cancer cells migrate, using diffusion MRI.

Some studies of cancer cell migration have suggested focal adhesion kinase (FAK) as a potential therapeutic target in solid carcinomas. FAK is an intracellular tyrosine kinase recruited to sites of integrin clustering or focal adhesions and is a multi-functional regulator of cell signalling within the tumour microenvironment. FAK is a major mediator of signal transduction by cell surface receptors including integrins, cytokine receptors and growth factors and could even potentially be used to inhibit cells migrating by adhesion. Based on preclinical evidence presented during this lecture, a FAK imaging biomarker would be useful to study this effect *in vivo* in combination with MRI sequences of apparent diffusion coefficient (ADC) (collagen distribution), pH and hypoxia, so as to better our understanding of the tumour microenvironment.

The tissue *macro*environment and its relationship to cachexia in cancer patients was also reviewed. Magnetic resonance spectroscopy (MRS) of lipids and cholesterol in serum has been shown to be prognostic for cachexia and builds on the idea of a tumour metabolic “secretome” in cachexia which could be used for prognostication and/or monitoring response to treatment using a holistic imaging approach with PET/MRI.

In light of recent advances in theranostic imaging, a role for PET/MRI was suggested looking at targeted prodrug detection and its action in tumours such as the conversion of 5-fluorocytosine (5-FC) to the active anti-cancer drug 5-fluorouracil (5-FU) in prostate cancer treatment, and the prospect of studying the effects of photo-immunotherapy [10]. These approaches go far beyond the capabilities of PET/CT and demonstrate potential unique future applications for PET/MRI.

## Dialogue Boards

### Dialog Board 1: Physics and Instrumentation

One of the main topics which has dominated discussions in past workshops has been the accuracy of attenuation correction (AC) using MR-based techniques. A number of strategies have been investigated, resulting in over 250 publications over the past several years. Attenuation due to the MR hardware alone can account for up to 20 % signal loss in the reconstructed PET image [11–13]. Nevertheless, there was general agreement between the participants in this Dialogue Board, and many clinical users, that this issue can be put to rest since MR-AC has been solved to the degree required for clinical use based on accepted image metrics. Sequences, such as ultra-short echo time (UTE) and zero echo time (ZTE), now provide sufficient information to identify bone or ancillary positioning aids and coils to aid in building an accurate attenuation map of the head for PET/MR neuroapplications [14]. Truncation of the body at the periphery due to the limited size of the MRI field-of-view can been mitigated with the B_0_ homogenization using gradient enhancement (“HUGE”) correction [15]; implementations of this type or alternative solutions vary between the manufacturers.

Of note, the variety of available algorithms for MR-based attenuation correction challenges the clinical readers. Unlike in PET/CT, where a single fast “push-button” whole-body CT scan provides all the necessary information required for AC, similar corrections in PET/MRI resemble a “LEGO construction kit”: multiple “building blocks” are needed to form a whole-body data set for attenuation correction in PET/MR: Dixon sequences for AC of the soft tissues, UTE and ZTE sequences to provide the skull bones, bone models to add major bones, HUGE or MLAA data to correct for truncation artefacts, and CT-based templates to correct for RF coils and the patient table. It is clear that this rather complex approach of PET/MR attenuation correction is prone to errors and requires thorough integration to provide the “push-button” ease of use, accuracy and robustness of the CT component for AC in PET/CT.

In light of the above discussions, it was agreed that although numerous methods for AC in PET/MRI have been developed and evaluated in various studies, not all of these different techniques have found their way as product versions into the existing PET/MRI systems. Thus, the PET/MRI community faces the situation that at single sites some methods are available, whilst others use different methods, different vendors and different AC applications. This leads to a certain inhomogeneity when using different AC methods, as not only *one* single version of AC exists as is the case for PET/CT. This also emphasises the further need towards standardisation efforts in PET/MRI attenuation correction.

The meeting was reminded, however, that the reproducibility of SUV_max_ measurements with [^18^F]FDG PET/CT can be of the order of up to ± 50 % as shown in a study where subjects were scanned on two consecutive days [16]. These differences are due to many factors including biological and instrumentation components, and small differences in attenuation correction factors between methods should be viewed in this context.

**Fig. 2.**
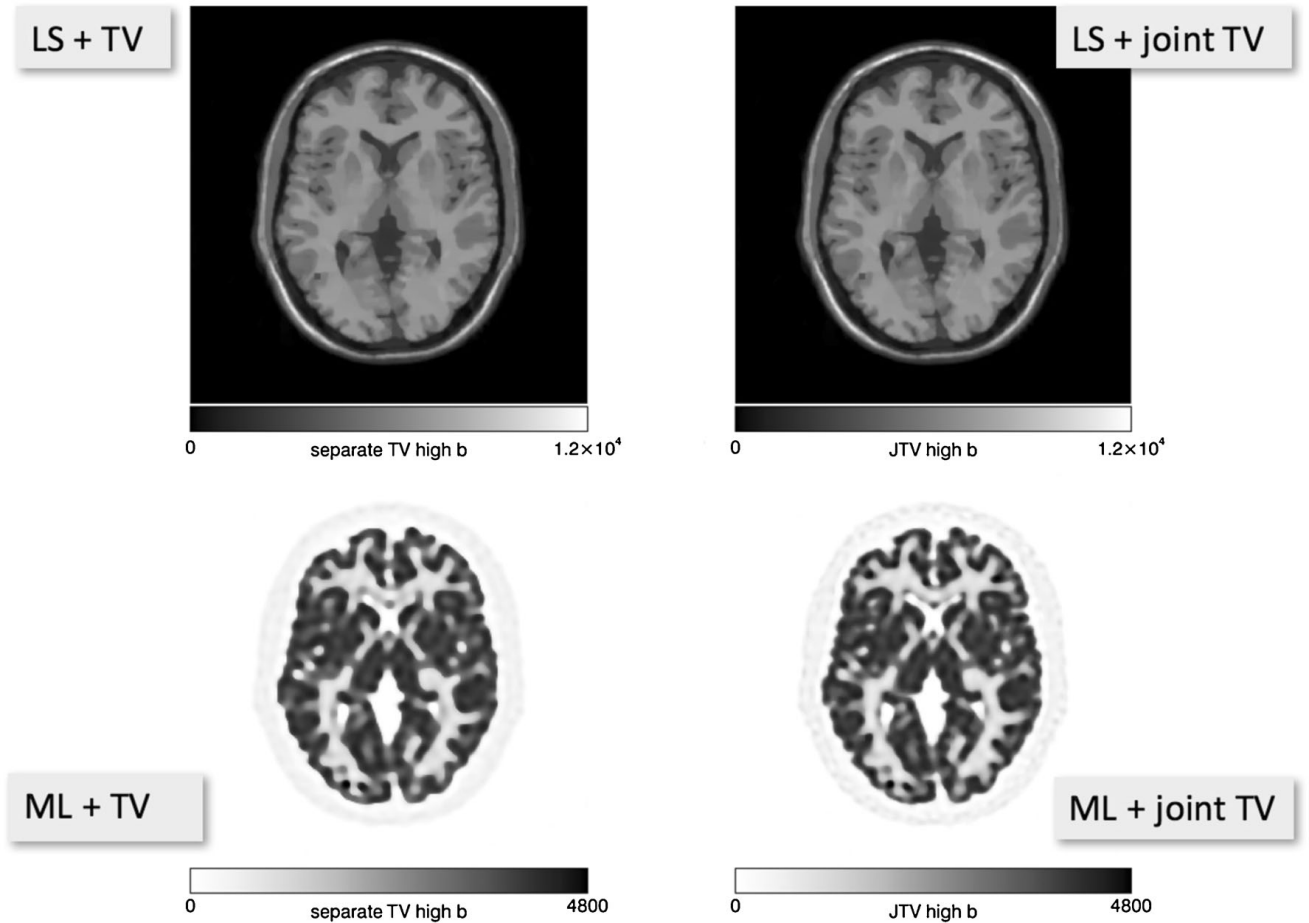
Joint PET and MR image reconstruction. Simulated PET and undersampled (5 of 8 coils) T1-w MR data. First independent reconstructions were performed, using least squares reconstruction with joint total variation (TV) prior to MR and ML reconstruction with a TV prior to PET. Then, a simultaneous MR and PET reconstruction with the joint TV prior was performed. The resolution of the PET image improves a little, whilst the changes to the MR image appear very minor at best (courtesy of J Nuyts, Leuven/BE).

The ability of PET data and MRI data to be used in the image reconstruction of the other modality (*i.e.* MR-informed PET reconstruction, PET-informed MRI reconstruction) has previously been suggested and explored [17]. The dialogue board concluded that using the MRI data can definitely have an impact and improve the spatial resolution and image quantification of PET images (Fig. 2), but the PET data have not yet provided any discernible improvement in the MRI reconstructions due to their poorer spatial resolution. Motion detected in the MRI scans can be used to correct for motion in both the MRI and PET data during the reconstruction process [14]. First implementations of these techniques are becoming available on commercial PET/MRI systems. Finally, one further comment on attenuation correction was added in that the accuracy of the attenuation correction could be further improved using time-of-flight information from the PET data, if available, in the MLAA algorithm [18–20]. This is now being actively pursued with the emergence of time-of-flight (ToF) PET/MRI systems. Yet, even without MLAA, improved image quality can be achieved by the use of improved ToF in regular ToF OSEM and it was shown that ToF already mitigates MR-based attenuation correction artefacts [21].

Table 3 summarises the progress made in the areas of PET/MRI physics and instrumentation. Of note, no such dedicated session was scheduled for the 4th (2015) or 5th (2016) workshops, when these topics were dealt with as part of the clinical topics. Overall, progress was made in all of the key areas. As discussed above, the accuracy and robustness of MR-AC methods are well understood, and research endeavors are now turning to the validation of new concepts for multi-parametric imaging, such as the adoption of an image-derived arterial blood input function [22].

**Table 3:** Progress of physics and instrumentation developments since the first workshop

Feature	2012	2013	2014	2015	2016	2017
Critical evaluation of MR-AC methods	↗	↑	↑	–	–	↑
Validation of MR-based motion correction	↔	↔	↗	–	–	↗
Agreement on acceptable lower limits of quantitative accuracy of PET following MR-AC	↘	↘	↔	–	–	↗
Clinical introduction of advanced, MR-based quantitative parameters (*e.g.* image-derived input functions)	↓	↘	↘	–	–	↗

### Dialogue Board 2: Oncology—*Status Quo*

The session started with a review of the current evidence for the role of PET/MRI in oncology (Table 4). From this, the areas where evidence exists that PET/MRI has been demonstrated to provide better information and characterisation of disease are in prostate cancer [23] and, as suggested previously at these workshops, in paediatric oncology [42]. Areas where new evidence is emerging include gastrointestinal cancers [43, 44], breast cancer [40, 41], gynaecological malignancies [37] and hepato-pancreatobiliary cancers [45]. The value of PET/MRI in these applications was seen again to be in a comprehensive regional evaluation and not in “whole-body” applications; as stated by one of the panellists: “The added value of PET/MRI starts locally”.

**Table 4:** Recent evidence on the role of PET/MRI in oncology, 2015 onwards (adapted from the presentation by V. Goh, London)

Tumour type	Studies	No. of patients	Comparator	Findings	First author [citation]
Prostate Ca ([^68^Ga]-PSMA)	1	66 (primary staging)	PET and multi-parametric MRI	Higher detection rate with PET/MRI	Eiber [23]
	1	119 (recurrence)	PET/CT	Higher detection rate with PET/MRI	Freitag [24]
Prostate Ca choline (^11^C or ^18^F)	1	31 (primary staging)	Multi-parametric MRI and FDG PET/MRI	Higher detection rate with PET/MRI	Lee [25]
Lymphoma	5	61 25 18 48 101	PET/CT PET/CT PET/CT PET/CT N/A	Comparable performance between PET/MRI and PET/CT	Hermann [26] Sher [27] Atkinson [28] Grueneisen [29] Kirchner [30]
Gastric Ca	1	42	ceCT	Higher accuracy than CT: 92.9 *vs* 76.2 % (reader 1) and 64.3 % (reader 2)	Ha [31]
Ca of unknown primary	2	20 43	PET/CT PET/CT	Outperforms PET/CT for detection of primary tumour	Ruhlmann [32, 33] Sekine [34]
Breast Ca (suspected recurrence)	1	36 (25 with recurrence)	MRI	PET/MRI: Sens 95 %, Spec 93 %, PPV 98 %, NPV 87 % MRI: Sens 82 %, Spec 86 %, PPV 94 %, NPV 63 % Higher diagnostic confidence with T1 + C VIBE	Grueneisen [35]
Mesothelioma	1	6	PET/CT	No difference between PET/MRI and PET/CT	Scaarschmidt [36]
Cervical Ca	1	27	Pathology (*n* = 20)	T stage; correct in 85 % Local invasion: Sens 82–100 %, Spec 90–100 % Nodal involvement: Sens 91 % Spec 94 %	Grueneisen [37, 38]
Oesophageal Ca	1	19	EUS, CT, PET/CT Pathology (*n* = 15)	T stage: correct in 66.7 *vs* 33.3 % (CT) and 86.7 % (EUS) N stage accuracy: 83.3 *vs* 50.0 % (CT), 66.7 % (PET/CT) and 75.0 % (EUS)	Lee [39]
Breast IDC	1	21	Pathology (IHC)	PET/MR biomarkers correlated with IHC phenotype in 13/21 patients (62 %; *P* = 0.001) ER/PR-tumours demonstrated higher Kep_mean_ and SUV_max_ than ER/PR+ tumours HER2-tumours displayed higher Kep_mean_, SUV_max_ and ADC_mean_ than HER2+ tumours	Catalano [40]
Endometrial Ca	1	36	Pathology	SUV_max_ and ADC_min_ increased in higher grade, advanced stage, deep myometrial invasion, cervical invasion, lymphovascular space and lymph node metastasis (*P* < 0.05)	Shih [41]

*Ca* cancer, *CUP* cancer of unknown primary, *ceCT* contrast-enhanced CT, *ER* estrogen receptor, *EUS* endoscopic ultrasonography, *IDC* invasive ductal carcinoma, *IHC* immunohistochemistry, *N* nodal stage, *NPV* negative predictive value, *PR* progesterone receptor, *PPV* positive predictive value, *PSMA* prostate-specific antigen, *Sens* sensitivity, *Spec* specificity, *T* tumour stage, *VIBE* volume-interpolated breath-hold examination

The dialogue board continued with a discussion of PET/MRI tissue characterisation and molecular diagnostics. It was felt that modern oncology practice is making its greatest advances today in DNA sequencing/phenotyping to guide the use of targeted therapies. This information can be transformed into knowledge about “tumour resistance”, which, it was suggested, is more likely attributable to clonal evolution producing heterogeneity [46]. This message was reinforced later in the symposium in the dialogue board on emerging areas and discovery (see below). PET/MRI was reported to be extremely valuable in avoiding sampling errors during biopsy caused by tumour heterogeneity [47].

Rather than trying to minimise the amount of scanning that would be performed, it was suggested that primary tumour characterisation for an individual might require multiple probes to characterise the individual disease; one example given was in the evaluation of primary lesions in hepatocellular carcinoma (HCC) using both [^18^F]FDG and [^18^F]fluorocholine (FCh). Whereas previously the value of [^18^F]FDG in HCC has been questioned due to the different glycolytic pathways that the tumour can access [48] leading to a negative [^18^F]FDG signal, it was argued that the best understanding of the tumour biology and heterogeneity would be attained by performing both scans to better understand the regional phenotype of the heterogeneous cancer cells. This is akin to the approach taken in PET imaging of neuroendocrine tumours using a somatostatin receptor radiopharmaceutical such as [^68^Ga]DOTATATE combined with [^18^F]FDG [49, 50].

The characterisation of HCC can be regarded as a molecular extension to the concept of *radiomics*, which has been applied primarily to anatomical image analysis in combination with non-imaging biomarkers. By integrating molecular imaging information with radiomics, we move to a “radiomics+” concept that is one step closer to “*in vivo* pathology”, which has the potential to become the standard in the near future for oncology patient management.

**Fig. 3.**
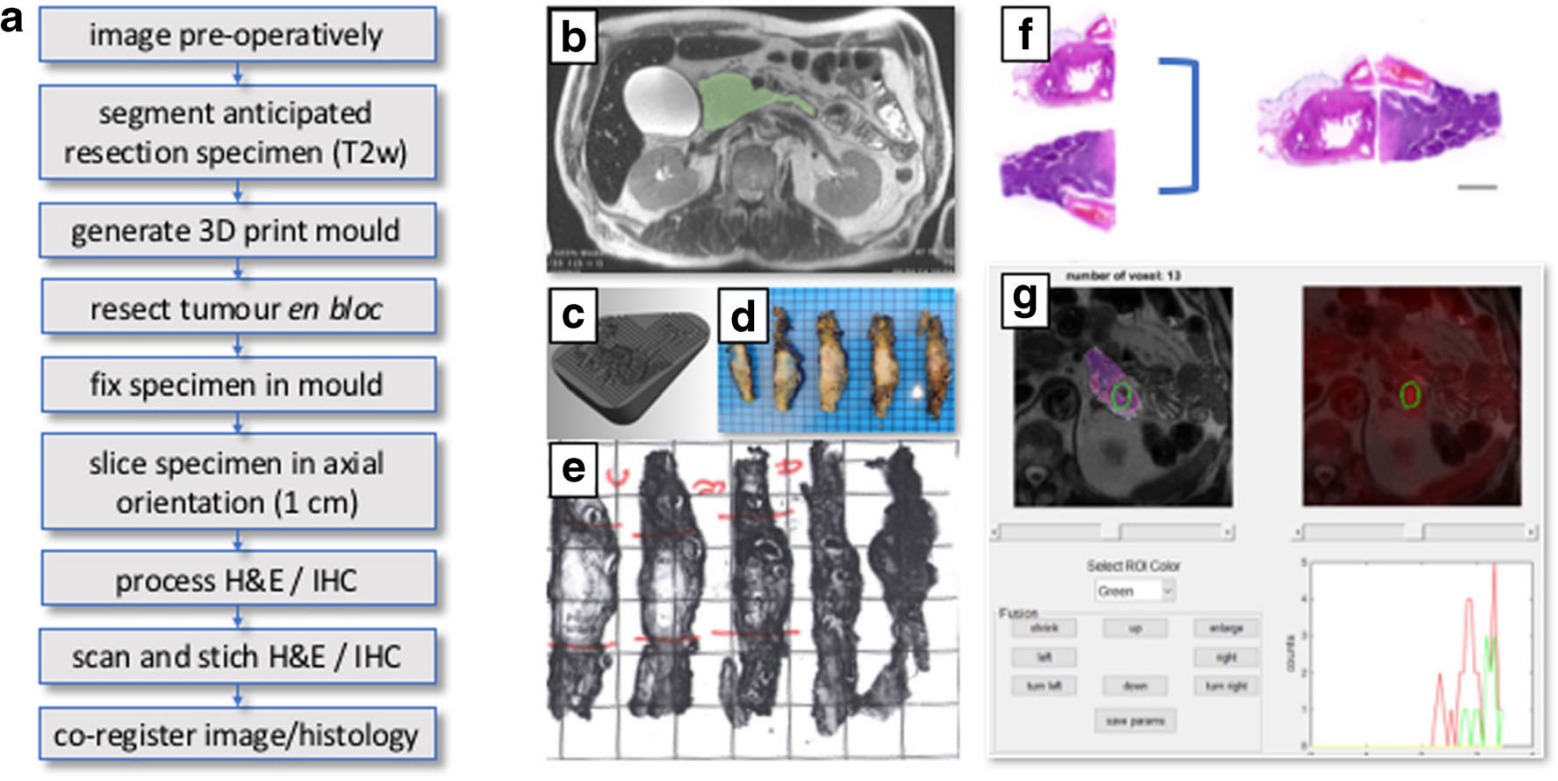
Co-registration of imaging and histopathology data. **a** Work flow schematic. **b** Pre-operative T2w image with annotated resection margins. **c** 3D print mold. **f** Fixated axially processed specimen with **g** annotated tissue blocks. **h** Stitching of tissue slices. **i** Screenshot of in-house written software for co-registration and regional analysis of imaging and histopathology data (courtesy of Rickmer Braren, Katja Steiger, Franz Irlinger and Maximilian Baust).

The theme of multi-parametric imaging and tumour heterogeneity continued in the presentations and discussions. In pancreatic adenocarcinoma, three distinct phenotypes with different genetic subtypes have been identified that exhibit different metabolic subtypes following either glycolytic or lipogenic pathways, or a combination of both [51]. A novel technique (Fig. 3) for spatially aligning surgical resection specimens with the images from PET/MRI was described using a histopathological processing algorithm based on a 3-D print mould generated from pre-operative *in vivo* imaging, enabling accurate co-registration between the excised tissue and the *in situ* imaging data. A multi-parametric analysis comparing [^18^F]FDG SUV *versus* ADC from MRI was used to classify heterogeneity and is a potential target area of application for ML-based clustering of physical tissue compartments.

Table 5 summarises the *status quo* of PET/MRI in oncology. Generally, the use of PET/MRI for oncology indications continues to grow and methodological progress has been made continuously. Unfortunately, the community still lacks a set of standardised acquisition protocols (pending the engagement of the medical specialists’ organisations), and further efforts are needed to resolve residual bias from MR-AC, the presence of truncation artefacts and, more importantly, motion effects.

**Table 5:** Progress indicators for PET/MRI in oncology

Feature	2012	2013	2014	2015	2016	2017
Definition of key clinical applications	↔	↔	↗	↗	↗	↗
Diagnostic quality of PET in PET/MRI equivalent to PET quality in PET/CT	↔	↔	↗	↗	↗	↑
Resolving quantitative bias from MR-AC	↘	↔	↔	↔	↗	↗
Clinical data available on diagnostic accuracy of PET(/MRI) in oncology	↔	↔	↗	↔	↗	↗
PET/MRI protocol standardisation	↓	↔	↘	↔	↔	↔
Clinical evidence on the usefulness of PET/MRI in paediatric oncology	↔	↗	↗	↗	↔	↔
Reduced radiation exposure as a key driver for paediatric PET/MRI	↗	↑	↑	↔	↘	↘

### Dialogue Board 3: Oncology—Where to Go?

This dialogue board addressed the theme of how PET/MRI can best complement the latest developments in oncology therapeutics. As immunotherapy is one of the hottest topics at present in oncology, discussion centred on whether it could be improved by the use of functional imaging techniques. Additionally, the discussants considered how the latest developments in relatively non-invasive tumour phenotyping using liquid biopsy could be combined with the results of imaging of lesion heterogeneity using PET/MRI. Liquid biopsy of circulating tumour cells and DNA or miRNA is playing an increasing role in screening and early detection (especially in recurrence), the detection of micro-metastases and in the monitoring of therapies where they can be used as an early indication of response from simple blood sampling [52, 53]. However, it was recognised that blood-based tests present a “global” picture as changes in phenotype are often observed serially, thus requiring regional identification, indicating an example of where liquid biopsy and imaging would perform a complementary role.

**Fig. 4.**
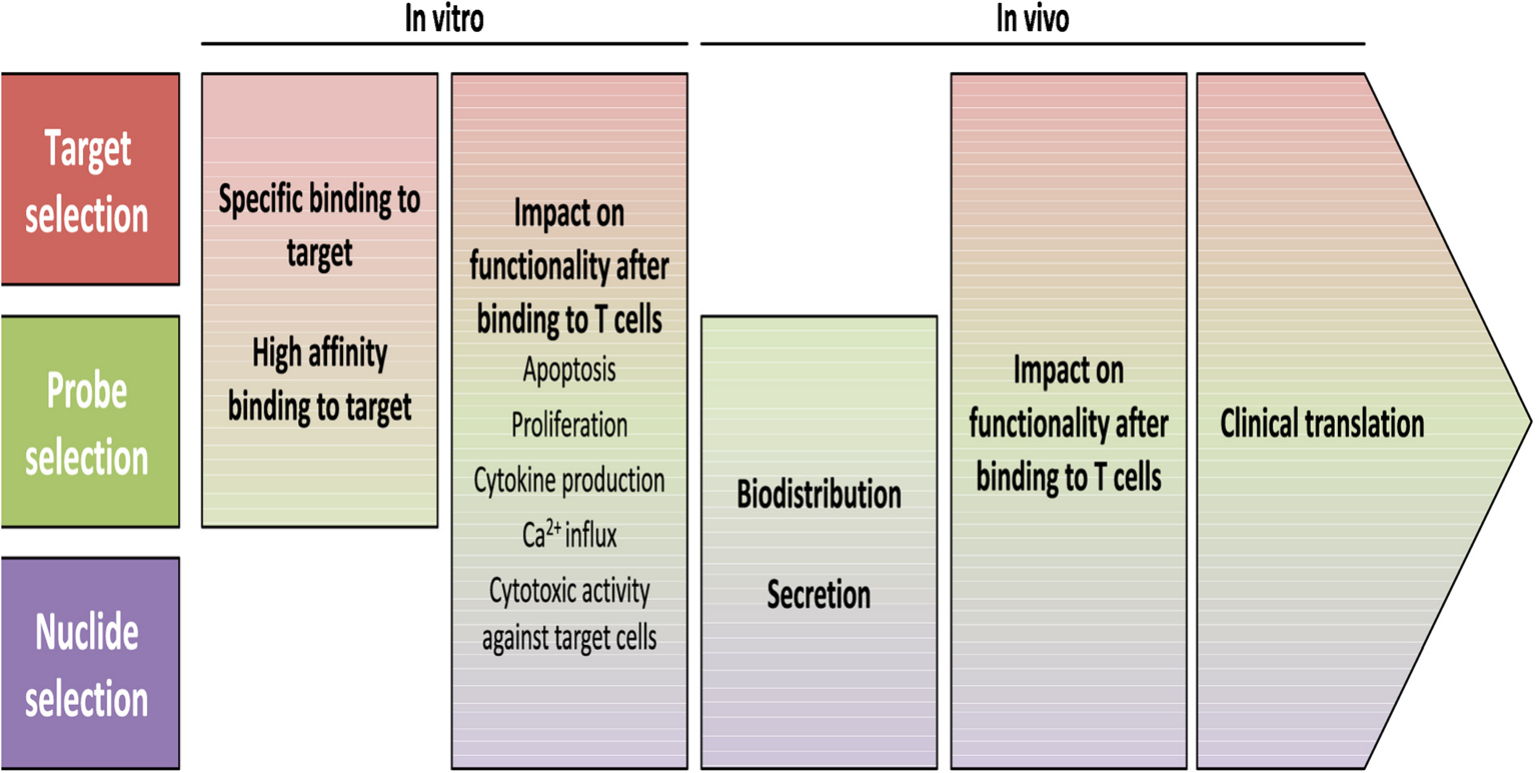
Selection of target structures on effector T cells as well as probes and nuclides to develop safe and efficient imaging strategies to track T cells during immunotherapy (courtesy of Sabine Mall and Angela Krackhardt)*.*

As this dialogue board was addressing the question of “*Where to Go?*”, the role of mouse models and patient-derived xenografts (PDXs) were discussed. Preclinical imaging with PET/MRI will likely have a role in these models along with bioluminescence techniques. Finally, the understanding of the role of tumour-infiltrating T cells was considered and whether labelling T cells with Zr-89 or other nuclides would allow *in vivo* monitoring to predict response to immunotherapy (Fig. 4). However, careful selection and matching of targets, probes and nuclides is required. Especially, functional impairment by direct targeting of T cells needs to be excluded.

### Panel Discussion: Dialogue Boards 2 and 3—Oncology

The discussion was kicked off with an upfront statement by one of the panellists who stated “the imaging field was disconnected from oncology for years”, thereby attesting to another statement below regarding a frequently observed approach towards interpreting results from imaging examinations in isolation. The panel was then asked to respond to the question of where they thought oncological therapy response assessment will be in 10 years’ time. The initial response was that there is increasing pressure to *predict* what response might be likely to occur in an individual patient, partly due to the high cost of the therapies. The power of predicting patient-specific therapy response was felt to be of ever-increasing importance given the evolution of resistance of solid tumours to successive lines of therapies. It was mentioned that 95 % of the oncology drugs fail during phase 1 trials, which can be regarded as an argument towards “raising the bar on pre-clinical testing”, *i.e.* questioning whether animal models are suitable surrogates for predicting efficacy in humans. It was felt that imaging, and molecular imaging in particular, will be an increasingly important tool due to the heterogeneity and evolution seen in disease progression and that ultimately imaging could be more important than traditional biopsy in this regard due to its ability to identify evolving clones. A more careful and systematic validation of multiple imaging parameters, as provided by PET/MRI, in the clinical context was considered an important step busting acceptance of imaging parameters as bio- and surrogate markers.

The panel was also asked to speculate as to the appropriate timing of imaging to assess response. After some discussion, with suggestions from “as soon as 24 hours” after commencing treatment to “not before 6–8 weeks”, it was agreed that if the therapy is inducing senescence [54] then imaging could be used as soon as 2 weeks after commencing therapy. One of the panellists suggested that we still do not have all of the appropriate tracers to study cellular biology to match the advances in oncology today. He suggested that some of the probes needed to demonstrate tumour stresses during therapy and the stress support pathways should be able to demonstrate apoptosis, senescence and hypoxia. The forum’s general response was that “we have the tracers and we have all the technology and tools, but what we do not understand is what the signal is telling us at present”. This returned to the theme of better developing multi-parametric analyses and the role that predictive analytics may play in this domain; one attendee summarised it by saying “we should stop talking about the “images”, but rather promote the image content as mineable data, that is, as an imaging assay”.

**Fig. 5.**
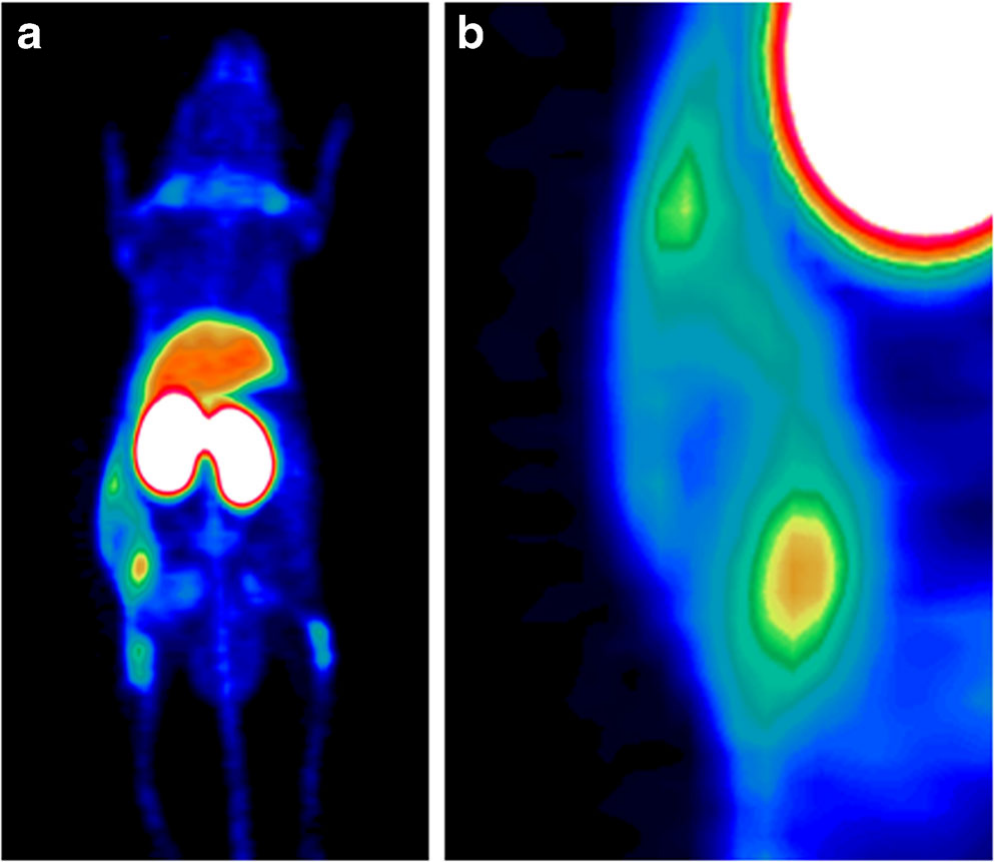
Tracking of intravenously injected T-cell receptor (TCR)-transduced central memory T cells within antigen-expressing tumours by Zr-89-labelled aTCRmu-F(ab′)_2_ using PET/CT. **a** TCR-transgenic T cells were injected intravenously after tumour engraftment followed by i.v. injection of [^89^Zr]-labelled aTCRmu-F(ab′)_2_ 48 h after adoptive T-cell transfer. **b** Heterogeneity of T-cell infiltration, as seen in the zoom in, has been validated by semi-quantitative analysis using immunohistochemistry [55] (courtesy of Sabine Mall and Angela Krackhardt).

The discussion moved on to looking at pseudo-progression in the light of immunotherapies, which is a real clinical challenge. Thus, imaging strategies need to be developed to directly track T cells within the tumour indicating response to treatment and therefore representing a valuable surrogate marker (Fig. 5). In addition, the ability to follow-up on treatment response and tumour progression with the help of liquid biopsies with or without imaging was discussed. It was concluded that, today, liquid biopsies alone would not be sufficient to do the job, and that imaging could add valuable information especially in therapy response monitoring providing information about the change in phenotype of known and the location of evolving (metastatic) disease which may have implications for subsequently adjusted therapies.

The panel discussion ended with some speculation about the role that functional imaging could take to assist in triaging patients appropriately to targeted therapies. It was felt that some education of regulators and the industry was still needed to develop a paradigm that would test individual patients prior to commencing therapy in an attempt to contain costs and produce better outcomes.

### Dialogue Board 4: Neurology—*Status Quo*

The dialogue board in neurology started by reasserting that the issue of AC for PET/MRI in neurology was solved to within the uncertainties associated with the overall procedure for the given modality. The only possible exceptions at this stage are in the cases of scanning children’s brains using atlas-based techniques for MR-derived AC or where there has been prior craniotomy and, therefore, disruption of the skull contour. This has now resulted in progress in analysing the data from brain PET/MRI, as previously researchers were preoccupied with solving the attenuation correction problem.

Presentations from the panellists documented the current clinical uses of PET/MRI in neurology as being in studies of dementia using [^18^F]FDG, neurodegenerative disorders, neuro-oncology, epilepsy, a small number of cases of cerebrovascular disease and various other diverse conditions including encephalitis and sarcoidosis [56]. The PET/MRI studies were being used in a variety of ways including diagnosis, characterising pathology, demonstrating the extent of neuronal injury and monitoring progression, and different radioligands can be used depending on the focus of the examination. The use of imaging biomarkers in Alzheimer’s disease (AD) with PET/MRI can be performed in a 20–30-min session including an early image after injection of the a fluorinated amyloid PET tracer to give a perfusion/“FDG-like” image (regional blood flow/metabolism) that can be compared with the subsequent image of the binding to cerebral amyloid. Mention was made of further imaging biomarkers such as the α4β2 nicotinic acetylcholine receptor which can be imaged using [^18^F]-flubatine [57]. Another promising approach to multi-modality brain imaging, particularly in dementia, utilises rest state-fMRI in combination with structural MRI and [^18^F]FDG PET to determine the functional connectivity between affected brain areas [58]. Finally, the use of PET/MRI in neurological imaging trials requires the judicious choice of an AC method (based on MR sequences or other modalities) that should stay standard for the duration of the study. Lastly, it was noted that fully integrated PET/MRI has proven to be beneficial for fully quantitative brain imaging, including non-/invasive pharmacokinetic modelling.

Discussion of research in rodents using PET/MRI focused on investigating the connectivity between metabolism (using [^18^F]FDG), perfusion (using [^15^O]H_2_O) and fMRI using the blood oxygen level dependent (BOLD) technique. A number of interesting results demonstrating both expected correlation in metabolism and perfusion in some brain areas as well as unexpected disconnections between the same parameters in other areas were seen [59]. PET/MRI, operating over the medium to long temporal scale compared to ultra-fast techniques methods such as electroencephalography (EEG) and magnetoencephalography (MEG), was seen as the only way to investigate such connections (termed “cometomics”—connectivity *via* metabolomics).

The great strength of PET/MRI in brain imaging, whether research or clinical, was thought to be the “one-stop shop” option, where all of the imaging required to characterise patient disease could be performed in a single session. This is also reflected in the continuous progress made in the applications of PET/MRI for neurology (Table 6). Of interest, one of the panellists made a point on the “simultaneity of PET and MRI, that had no other advantage than increase patient comfort and convenience”, which may have come as a surprise to some PET/MRI users.

**Table 6:** Progress indicators for PET/MRI in neurology

Feature	2012	2013	2014	2015	2016	2017
Improved understanding of brain physiology and function through the use of combined PET/MRI	↔	↗	↗	↗	↗	↗
Methodological progress for improved quantification of PET/MRI neurological examinations (AC, IDIF, SUV)	↔	↔	↗	↗	↗	↗
MR-based motion correction for routine clinical use	↓	↘	↔	↔	↔	↗

### Dialogue Board 5: Neurology—Where to Go?

This dialogue board posed the question of whether the predominant use of brain PET/MRI in the future would be for routine clinical applications or in research. Ample evidence was provided that there is an increasing role clinically for brain PET/MRI [6]. The case was made for the substitution of imaging biomarkers to objectively diagnose dementia in place of the current syndromic tools which clinicians are left to rely upon. With potentially expensive therapies on the horizon in AD, it was felt that regulators and medical service providers should be encouraged to use imaging as an objective biomarker to select the appropriate patients for these therapies as well as to monitor response.

On the research side, elegant work using multi-modality neuroimaging with pharmacological challenges was shown which demonstrated that pharmacological PET could be used to study drug penetration and kinetics, to identify pharmacodynamic effects, and could be used in drug occupancy studies which could be combined with simultaneous blood flow measurements using arterial spin labelling [60] as well as functional and structural MR imaging [61, 62].

Addressing the issue of where to go with future brain PET/MRI studies, the integration of imaging with human genetics testing was explored. Using next-generation sequencing technologies, it is now possible to sequence hundreds and even thousands of genes in parallel. This dramatically increases the chance to find the cause of the disease in many heterogeneous neurologic diseases. From a diagnostic point of view, panels of genes that are known to cause a particular disease including all differential diagnoses can be investigated simultaneously with very high coverage and, if negative, whole exome or whole genome sequencing is followed. Next-generation sequencing allows also the detection of mosaicism in blood and other tissue samples.

### Panel Discussion: Dialogue Boards 4 and 5—Neurology

The panel discussion on the neuroscience subjects reflected the relative maturity and acceptance of PET/MRI in studying the brain. The panellists agreed that PET/MRI is a convenient means to an efficient work-up of patients suspected of Alzheimer’s disease; however, the added diagnostic benefit of fully integrated PET/MRI in this patient group was considered to be small.

PET/MRI can help increase patient comfort as well as provide doctors with the option to perform a wide range of structural and molecular imaging assessments for disease characterisation and/or monitoring within a single investigation. This is of utmost importance for trials comprising a variety of MRI/fMRI imaging sequences along with extensive and complex pharmacokinetic PET investigations. The panellists agreed that PET/MRI is capable of providing molecular imaging-based evidence to support early diagnosis/staging/disease and therapy monitoring of dementia and other brain disorders in clinical routine. Furthermore, it is a powerful tool in complex research settings of neuroscience comprising molecular imaging and pharmacokinetic/pharmacodynamic analysis.

In clinical research, a large number of physiologically relevant measurements can now be made simultaneously using PET and MRI including metabolism, neurotransmission, receptor expression, cerebral blood flow/perfusion, tissue environment and pathological conditions (*e.g.* amyloid deposition). This opens for investigation of fundamental physiological and neurochemical aspects of, *e.g.* changes in neurotransmission in relation to blood flow under physiological or pharmacological stimulation. Such knowledge can be instrumental to assess drug effects in the individual patients, and allows for a precision medicine approach. The next challenge seen by the panellists was how to integrate these measurements with genetic fingerprinting to delve more deeply into describing a more comprehensive phenotypical classification of disease.

An additional emerging topic might also be the assessment of active plaques in demyelinating diseases like multiple sclerosis. In addition, the combination of PET/MRI with fast temporal scale techniques like EEG might give further insight in pathophysiological processes in the brain, *e.g.* in epilepsy.

### Dialogue Board 6: Infection and Inflammation

The topics of infection and inflammation imaging were introduced at last year’s workshop for the first time [5]. At the time, an enormous number of targets were presented for potentially imaging infection and inflammation, but there were relatively few imaging biomarkers specifically developed to exploit the vast majority of these, and hence discussion last year centred on adapting well-established nuclear medicine techniques which have been around for many years using radiolabelled antibiotics, immune cells and antibodies, membrane ligands, antimicrobial peptides, iron metabolism ([^67^Ga]-citrate) and metabolic tracers. However, many existing agents fall short of distinguishing infection from sterile inflammation (*e.g.* FDG). In contrast, at the meeting this year, a number of new compounds were presented for bacteria-specific PET imaging. Non-invasive anatomical analysis with MRI plus pathogen-specific PET imaging could significantly improve patient outcomes by rapidly identifying a source of infection and monitoring the response to treatment [63].

Imaging of infection has been developed by the Center for Infection & Inflammation Imaging Research (Ci3R) at Johns Hopkins University over the past few years. Using a systematic screening approach, they evaluated a large number of potential agents for selective bacterial accumulation and found ten compounds that could be used as bacteria-specific imaging biomarkers, showing interesting results with *para*-aminobenzoic acid (PABA), deoxy-mannitol and deoxy-sorbitol [64]. Among other biomarkers, they presented results using [^18^F]fluoro-deoxysorbitol ([^18^F]FDS), which can be synthesised from radiolabelled FDG [65]. [^18^F]FDS is selectively accumulated by gram-negative Enterobacteriaceae such as *Escherichia coli*, *Yersinia*, *Klebsiella*, *Enterobacter* and *Salmonella*, including multi-drug-resistant organisms. Using a murine myositis model, [^18^F]FDS was able to differentiate infection sites from sterile infection in immunocompetent and neutropenic mice, thus suggesting that the uptake is actually by the bacteria rather than by migrating neutrophils. Potential advantages of imaging active infection include a much earlier path to diagnosis and instituting appropriate antibiotic therapy rather than waiting for blood cultures (Table 7).

**Table 7:** Progress indicators for PET/MRI in infection and inflammation

	2012	2013	2014	2015	2016	2017
Improved tissue characterisation by combined PET/MRI	–	–	–	–	↗	↗
Development of new radiopharmaceuticals for PET use in general	–	–	–	–	↗	↗
Standardise imaging protocols	–	–	–	–	↔	↔
Standardise image interpretation criteria	–	–	–	–	↔	↔
Definition of key clinical applications	–	–	–	–	↗	↔

The session then turned to a new area for the workshop: the imaging of chronic pain and how its investigation could benefit from the use of [^18^F]FDG and PET/MRI. In many Western societies, morbidity and productivity loss due to chronic pain constitutes one of the largest burdens on society. Chronic pain was said to affect more people than those suffering from cancer, heart disease and diabetes combined. The source of chronic pain is often difficult to identify and diagnose with conventional morphological imaging. Bone scanning using [^99m^Tc]phosphonate has long been known to have a role in identifying the site of the cause of pain reflected in increased osteoblastic reactivity—the concept of “where it’s hot it hurts” attributed to Schuster. Similarly, increased PET radiotracer uptake preliminarily appears to map to areas of increased pain-relevant or pain-generating pathology, potentially affording clinicians the ability to make improved image-informed, objective management decision to minimise pain in chronic pain sufferers. The MRI component of PET/MRI is considered essential because of the high spatial resolution and high tissue contrast which, when combined with the [^18^F]FDG signal, can identify the exact location of the inflammatory response. As soft tissue is involved in many of these syndromes, PET/CT was thought to be of less value due to the poorer tissue contrast that is usually seen with CT compared to MRI. Additionally, because PET and MRI data sets are acquired simultaneously, the fidelity of the co-registration of both data sets is likely more accurate than what can be achieved with PET/CT. Spatial mis-registration between PET and CT data is a well-known phenomenon since both data sets are acquired separately in time and, thus, small patient movements between the two scan acquisitions can result in PET measurement errors between small adjacent structures such as a nerve root and a neighbouring facet joint. The ability to accurately delineate abnormal radiotracer uptake in a nerve root *versus* the facet joint, for example, could be the difference in a successful outcome since management decisions are made according to the location of PET abnormality.

[^18^F]FDG has been used off-label in a clinical trial to study patients with complex regional pain syndrome, chronic sciatica and other pain syndromes. As in the discussion of infection and inflammation imaging, new imaging biomarkers of specific targets were being developed and characterised in human subjects, for example, to identify sigma-1 receptor (σ1R) ligands such as [^18^F]FTC-146 in the setting of chronic pain [66, 67]. Sigma-1 receptors, a unique class of intercellular chaperone proteins, have a modulatory role in ion channels and other neurotransmitter systems. Accordingly, σ1Rs have been found to be important in pain, inflammation, neuronal protection, neurodegeneration, cancer, addiction and psychiatric diseases. Early results have showed important differences between asymptomatic volunteers and those suffering from chronic pain.

### Dialogue Board 7: Emerging Areas

The final dialogue board of the workshop addressed the issue of advances in multi-parametric imaging. The first contribution looked at how it is now possible to investigate multiple aspects of tumour metabolism by combining hyperpolarised C-13-labelled cell substrates with PET biomarkers, such as [^18^F]FDG [68, 69]. Dynamic nuclear polarisation can increase the signal-to-noise ratio in solution state ^13^C NMR spectroscopy and imaging experiments by >10,000× [70]. Fundamental questions such as why cancers have high rates of aerobic glycolysis (Warburg effect) could potentially be addressed using 1-[^13^C]pyruvate and [^18^F]FDG in animal tumour models. The combination of magnetic resonance spectroscopic imaging of hyperpolarised 1-[^13^C]pyruvate, when combined with [^18^F]FDG to image glycolysis, and further measures such as hypoxia biomarkers, could provide insight into cancer metabolism and tumour heterogeneity and how various pathways (*e.g.* HIF1α, MYC, mTOR, PTEN) are involved (Table 8).

**Table 8:** Progress indicators for PET/MRI for applications in emerging areas

	2012	2013	2014	2015	2016	2017
Fully integrated PET/MRI exclusively offers the largest variety of multi-parametric biomarkers	↔	↗	↑	↑	↑	↑
Validation of advanced multi-parametric biomarkers in clinical research (beyond “image fusion”)	↘	↔	↗	↗	↔	↔
Contributions of small animal imaging to the understanding of multi-parametric biomarkers	↔	↗	↑	↑	↗	↗
Using standardised approaches for assessing the accuracy of PET/MRI and towards multi-parametric image analysis	–	–	–	↗	↔	↔

The second contribution was on the development of adaptive therapies and insights into how cancers “evolve”. Evolution is driven by the genomic plasticity that is inherent to cancers, in combination with microenvironmental selection. As tumours contain multiple microenvironmental habitats, these will result in distinct lineages of tumour cells generating intratumoural heterogeneity. Addition of therapy changes the adaptive landscape and selects for cells that are resistant. Notably, these resistant clades of cells are often cross resistant to other therapies, leading to unmanageable disease [71]. This has been mathematically modelled to show that emergence of resistance can be forestalled with adaptive dosing based on tumour response [72]. This is highly relevant to PET/MRI studies as the monitoring of response becomes a critical factor in adaptive dosing and in predicting pathways that the emerging resistant cell lines may exploit. The large number of probes that can be used with PET and MRI should provide tools with which to monitor the differential response expected from the different phenotypes of cancer cells present.

The final presentation looked at a promising technique for MRI, known as chemical exchange saturation transfer (CEST) [73, 74] with glucoCEST, which uses the hydroxyl groups of the glucose molecule to study glucose uptake and metabolism [75]. The glucoCEST imaging procedure uses millimolar amounts of contrast and demonstrates vascular and extracellular signals and may potentially be able to follow metabolic products of the agent through various phases of the Krebs cycle. This could provide an interesting comparison with [^18^F]FDG, which is trapped upon entry into the cell and does not proceed through the metabolic cascade. A further agent which has already been tested in tumour patients is 3-O-methyl-D-glucose (OMG) [76–78]. Of note, chemical shift selective CEST, *e.g.* amide proton transfer, works best with very high magnetic field strengths and, thus, is not applicable with current whole-body PET/MRI systems. However, the glucose injection approach employing the difference of images before and after injection again provides glucose-related selectivity at clinical field strengths and lower chemical shift selectivity.

This dialogue board brought out some of the liveliest discussions, attesting to the keen interests of the audience in reviewing new, less standard-of-care applications of PET/MRI. A strong desire towards implementing existing MR-only techniques (*e.g.* CEST imaging, hyperpolarisation) into PET/MRI was expressed. Whilst progress in conceptualisation of such ideas was evident, practical implementations were lagging behind, primarily because these implementations are linked to higher investments in personnel and infrastructure, and as such may be limited to a few selected sites. In that case, it becomes ever more important to share new insights into emerging areas in order to ensure expedited translation into the clinic where and whenever applicable.

## Round Table Discussions

### Round Table 1: Reading PET/MRI—Who and How?

Round Table 1 hosted a spirited debate about the best way to report clinical PET/MRI scans. The panellists represented the radiological, nuclear medicine and hybrid imaging communities. From the opening remarks of each, it was clear that this issue is one that had been prominent in the panellists’ minds for some time.

The majority of the panellists introduced PET/MRI in their institutions with clinical scanning reported by dual readers—an MRI expert and a nuclear medicine expert. This was seen as a sensible approach in the initial start-up phase, but some felt that this would be financially unsustainable in the long term. All agreed that it was imperative that the potential of MRI should be fully exploited and not “dumbed down” as some panellists suggested happened to the CT component of PET/CT whenever its use is limited to “low dose” mode for anatomical localisation and attenuation correction only.

Having accepted that dual reporting was not the future for PET/MRI in the long term, the debate moved to whether a dual certified radiology/nuclear medicine-trained individual is appropriate or, in fact, whether a new specialisation as a “hybrid imager” would be more appropriate. The hybrid imager would be someone particularly trained in the interpretation of multi-parametric image data in an integrated, synergistic fashion, rather than as a pair of complementary scans acquired in a single session. Training programmes will need to be modified to reflect any such changes in specialisation which, unfortunately, still happen at a national level even within the European Union. The respective professional organisations will need to cooperate to produce the best outcome and not revert to trying to simply “protect their patch”.

Eventually, the healthcare environment will need to accept the statement made by one of the panellists: “High-end technology imaging requires high-end reading”; local variations may apply.

### Round Table 2: Imaging *Versus* Liquid Biopsy

The participants in this round table discussion emphasised that liquid biopsy today is able to characterise multiple mutations that may not be present in every individual lesion and, therefore, is not prone to the sampling errors associated with conventional tissue biopsy using fine needle aspiration or core biopsy samples. From this point of view, it was suggested that it was an extremely valuable technology for monitoring the development of resistance in cancers. Liquid biopsy may point to the use of different imaging biomarkers if multiple driver mutations are demonstrated in the sample. Panellists pointed to the massive imbalance between the relatively modest amount spent on diagnostic techniques compared with the high cost of the therapies that are often delivered without adequately characterising the most appropriate pathway to target prior to treatment. One panellist called for “spending more money on tests that help us understand disease and tailor much more expensive therapies.”

It was clear from the discussion that liquid biopsy and imaging are complementary rather than competing technologies. Liquid biopsy is able to identify different phenotypes in cancer cells but remains a global measure, whereas imaging with PET/MRI using appropriate imaging probes can be used to identify differences between lesions due to clonal variation which may have an influence on the choice of the most appropriate therapy, especially when using a regionally targeted approach such as with radiotherapy or organ-specific chemotherapy or radioembolisation.

The discussion diverted multiple times onto a topic that surfaced repeatedly during this workshop, which is the wealth of data that we have at hand, augmented by imaging-based biomarker information, and that generally goes untouched. The panel argued that “even if we do not interpret all information [aka data] today, we may need these data for later”, pointing to the ever-evolving field of big data and deep learning. Such a data repository would clearly benefit from wrapping any type of non-imaging (and imaging) data in a structured, DICOM-type manner so as make them accessible on existing viewing and storage systems, such as PACS.

## Conclusion

In continuation of the discussions of the 5th Tübingen workshop, this meeting has focussed much more on emerging areas and provided a balance between state-of-the-art practices and strategies (“Where to go?”) in key areas of application (oncology and neurology). The dialogue boards were complemented with highlight presentations of promising applications of PET/MRI, such as RTP and the assessment of the tumour microenvironment, an area that clearly would benefit from cross-speciality engagement much beyond that required for operating a PET/MRI programme.

Two interesting take-home messages from this year’s meeting pertinent to the clinical community were as follows: (a) PET/MRI is perhaps better used as a local tumour phenotyping methodology rather than as a competitor to whole-body PET/CT imaging (except for paediatrics), and (b) the simultaneity of PET and MRI as offered by fully integrated PET/MRI systems is seen as useful mainly for increased patient comfort—for the actual use of PET and MRI information, maximisation of stand-alone performance and quality was understood as key.

In the summary review talk, the theme given to this year’s workshop was “global warming”. We have come a long way from the 1st workshop in 2012 when the topic was mainly “how does PET/MRI compare with PET/CT”. Over the years and through the engagement of many avid users and pioneers, the potential and capabilities of PET/MRI have been shaped and are becoming much more apparent. Together, the PET/MRI community has created a space for engagement of many capable individuals and groups. A global team effort is of the essence in turning this potential into a reality with real value for the research community and for patients. Global warming was chosen, because like climate change, PET/MRI is now known to many people. It has gone global and it has started to warm people up to its use.
